# Modification Effects and Mechanism of Cement Paste Wrapping on Sulfate-Containing Recycled Aggregate

**DOI:** 10.3390/ma18153617

**Published:** 2025-07-31

**Authors:** Xiancui Yan, Wen Chen, Zimo He, Hui Liu, Shengbang Xu, Shulin Lu, Minqi Hua, Xinjie Wang

**Affiliations:** 1 Department of Civil Engineering, Changzhou University, Changzhou 213164, China; yanxc@cczu.edu.cn (X.Y.); cw1375688465@outlook.com (W.C.); 15195385025@163.com (Z.H.); liuhui@cczu.edu.cn (H.L.); s24210814015@smail.cczu.edu.cn (S.X.); sanhui622988511@163.com (S.L.); 2 School of Civil Engineering & Architecture, Wuhan University of Technology, Wuhan 430070, China; hmq@whut.edu.cn

**Keywords:** sulfate-containing recycled aggregate, cement paste wrapping, modified recycled aggregate, water–binder ratio, fly ash

## Abstract

The utilization of recycled concrete aggregate presents an effective solution for construction waste mitigation. However, concrete service in sulfate environments leads to sulfate ion retention in recycled aggregates, substantially impairing their quality and requiring modification approaches. A critical question remains whether traditional recycled aggregate modification techniques can effectively enhance the performance of these sulfate-containing recycled aggregates (SRA). Cement paste wrapping in various proportions was used in this investigation to enhance SRA. The performance of both SRA and modified aggregates was systematically assessed through measurements of apparent density, water absorption, crushing value, and microhardness. Microstructural analysis of the cement wrapping modification mechanism was conducted by scanning electron microscopy coupled with mercury intrusion porosimetry. Results revealed that internal sulfate addition decreased the crushing value and increased the water absorption of recycled aggregates, primarily due to micro-cracks formed by expansion. Additionally, the pores were occupied by erosion products, leading to a slight increase in the apparent density of aggregates. The performance of SRA was effectively enhanced by cement paste wrapping at a 0.6 water–binder ratio, whereas it was negatively impacted by a ratio of 1.0. The modifying effect became even more effective when 15% fly ash was added to the wrapping paste. Scanning electron microscopy observations revealed that the interface of SRA was predominantly composed of gypsum crystals. Cement paste wrapping greatly enhanced the original interface structure, despite a new dense interface formed in the modified aggregates.

## 1. Introduction

Massive volumes of building and demolition waste are produced by the growing construction sector, which presents serious economic and environmental problems [1,2]. According to global statistics, almost 3 Gt of building waste is produced annually worldwide, yet only an average of 30% undergoes recycling processes [3]. One promising approach to resource conservation and sustainable building is to recycle this waste into recycled coarse aggregates (RCA) for use in recycled concrete. However, its mechanical qualities and long-term durability are frequently endangered by the inherent disadvantages of RCA, which are mostly caused by the adhered old mortar [4,5]. These disadvantages include higher porosity and lower density in comparison to natural aggregates. Therefore, research into efficient modification methods targeted at improving the performance characteristics of RCA has become a crucial and active topic of exploration in order to fully achieve the potential of building waste and enable its widespread, stable application in structural concrete.

Numerous investigations into strengthening recycled aggregate have been conducted. Three types of strengthening techniques for recycled aggregate are available: physical, chemical, and microbiological strengthening methods. Among physical strengthening techniques, grinding and scrubbing are the most prevalent and operationally effective approaches for removing adhered old mortar from RCA [6]. The combined high-temperature (<400 °C) and ball milling treatment significantly improved recycled concrete characteristics (7 d compressive strength reached 50 MPa), although it cost more than the single-ball milling approach [7]. Microwave pre-treatment was employed by Bru et al. [8] prior to mechanical grinding to significantly weaken the interfacial bonding strength of aggregates. This combined approach demonstrates superior sample fragmentation efficiency and reduces attached mortar. Chemical strengthening typically involves immersing recycled aggregates in chemical solutions to dissolve and remove the adhered old mortar from their surfaces. For instance, Tam et al. [9] treated recycled coarse aggregates with acidic solutions (e.g., HCl and H_2_SO_4_), which effectively improved their performance by stripping away the residual mortar. Al-Bayati et al. [10] evaluated the efficacy of thermal and chemical treatments for recycled aggregate enhancement, demonstrating that combining heat treatment (350 °C) and acid immersion significantly removed the attached mortar and improved the performance characteristics of RCA. Moreover, it is also possible to fill holes and micro-cracks with cement paste, polymer emulsion, sodium silicate solution, and nanomaterial paste to improve the structural integrity of recycled aggregates. Moreover, carbonation is a promising chemical modification method capable of CO_2_ sequestration [11]. Theoretical calculations indicate that 1.0 t of mortar in RCA can absorb approximately 0.5 t of CO_2_, demonstrating its potential for carbon-negative construction materials. Zhang et al. concluded that carbonation was beneficial to reducing water absorption and crushing value, but it had little effect on increasing the apparent density [12]. Microbial enhancement facilitates calcium carbonate deposition via surface adsorption and mineralization induction mechanisms, effectively enhancing pore structures of the aggregate [13]. According to Duan et al. [14], the highest healing width of microbial augmentation was 0.27 mm in RCA. Tang et al. [15] also proved that the performance of recycled concrete was effectively enhanced by a microbially modified approach.

Cement paste wrapping is an additional common method for strengthening RCA since cement raw materials are readily available and compatible with the concrete matrix. Cement hydrates to form Ca(OH)_2_ and C-S-H gel, which has a specific strength and serves as reinforcement [16]. Zhao et al. [17] found that RCA performed best when using a cement paste (w/c = 0.8) at an ideal thickness of 0.035 mm. The improvement functions were shown to be diminished by both excessive and insufficient coating thicknesses. The wrapping technique developed by Wang et al. [18], utilizing an effective cement-fly ash paste (w/c = 0.5, 20% fly ash), offered frost resistance improvement for concrete short columns. Contrary to expectations, Wang et al. [19] demonstrated that varying water–binder ratios (0.7–1.0) in paste coatings showed limited effects on RCA properties, while significantly enhancing mechanical properties of the corresponding recycled concrete. Optimal recycled concrete performance was achieved at a 0.9 water–binder ratio. The contradictory effects observed in modified RCA necessitate systematic investigation into optimizing the cement paste wrapping proportions, which holds significant theoretical and practical importance.

However, the service environment of parent concrete involves less study on recycled aggregate and concrete nowadays. The author explored and found that the aggregate performance of fatigued concrete was far inferior to that of the original aggregate [20]. Liu et al. [21] used carbonation technology to modify freeze–thaw damaged recycled aggregate. Sulfate attacks have seriously damaged a number of bridges and roads in the saline–alkali land and marine areas in China [22,23]. The concrete structure cracks and its service life is diminished when SO_4_^2−^ diffuses from the service environment into the concrete and reacts with cement hydration products to create gypsum and ettringite [24,25]. More seriously, when the concrete buildings in these locations are demolished, they frequently turn into sulfate-containing recycled aggregate (SRA) that is hard to utilize due to the presence of SO_4_^2−^ and sulfate attack products in the adhesive mortar of the recycled aggregate. This results in an internal sulfate attack if applied to the recycled concrete construction. Additionally, it may intensify the weakening effect of the interface between the cement mortar matrix and recycled aggregate [26]. Whether current modification approaches for traditional RCA improve the quality of SRA necessitates additional research.

In the present study, traditional RCA was obtained by crushing concrete of varying compressive strengths (C20, C30, and C40). GB/T 25177-2010 [27] limits the sulfate content in recycled aggregates to below 2% for structural concrete applications. This study aims to develop enhancement methods for recycled aggregates containing 2% sulfate to enable their utilization in structural engineering. Therefore, SRA was obtained by crushing concrete containing 2% sodium sulfate. The cement paste wrapping method was used in an effort to enhance the quality of traditional RCA and SRA. The aggregate characteristics, including apparent density, water absorption, and crushing value, were used to assess the modification effect. Microhardness and microstructure were analysed to explore the modification mechanism of cement paste wrapping.

## 2. Research Significance

Utilizing recycled aggregates effectively reduces the exploitation of natural resources in engineering projects while contributing to the reduction in environmental pollution brought by construction waste. However, recycled aggregate exhibits inferior performance compared to natural aggregate, primarily due to the inevitable adherence of old mortar from the parent concrete. Thus, implementing effective measures to enhance the properties of waste concrete recycled aggregate is crucial to ensuring its reliable application. In specific, where the parent concrete is exposed to sulfate conditions, the inherent sulfate ions in recycled aggregates will inevitably exert negative impacts on the aggregates themselves [26,28]. Cement paste wrapping can enhance the quality of recycled aggregates to some extent; however, the available previous literature has yielded inconsistent results regarding the optimal proportion of wrapping paste. For instance, Zhao et al. [17] identified the optimal water-to-binder ratio as 0.8, whereas Wang et al. [18] reported it as 0.5. Furthermore, it remains unclear whether paste wrapping can effectively enhance the quality of sulfate-containing recycled aggregates. Accordingly, our research objective is to identify the optimal paste wrapping proportion that is applicable to the modification of sulfate-containing aggregates. This aspect has not been adequately investigated in the prior literature. For this purpose, sulfate was initially incorporated into the parent concrete, which was subsequently crushed to prepare sulfate-containing recycled aggregates, facilitating the study of how internal sulfate impacted their physical and mechanical properties. Secondly, sulfate-containing recycled aggregates were treated using different paste wrapping proportions, and the effect and mechanism of cement paste wrapping on improving the quality of sulfate-containing aggregates are investigated through exploratory tests.

## 3. Materials and Methods

### 3.1. Materials

Parent concrete (C20, C30, and C40) was prepared using the following constituent materials: Portland cement with 42.5 grades, water, natural fine aggregate (apparent density of 2500 kg/m^3^, bulk density of 1450 kg/m^3^, fineness modulus of 2.4), and natural coarse aggregate (limestone, apparent density of 2600 kg/m^3^, bulk density of 1640 kg/m^3^, with a maximum nominal size of 20 mm). In addition, appropriate polycarboxylate superplasticizer (SP) was used to treat the fresh concrete mixture to improve the workability. The chemical composition of Portland cement and fly ash is shown in Table 1. It should be noted that fly ash was not incorporated into the parent concrete; instead, it was used in the paste wrapping process.

### 3.2. Parent Concrete

Three natural parent concrete grades (C20, C30, and C40), with varying strengths, were created for this investigation. By adding 2% sodium sulfate to natural concrete, C20S, C30S, and C40S, three types of sulfate-containing concrete were also created. Table 2 shows the specific proportions of each concrete type. It should be noted that the optimal method to simulate sulfate-induced concrete erosion is to expose the concrete to a sulfate-rich environment in the field. Due to time constraints, an accelerated approach was adopted to produce sulfate-containing recycled aggregates by incorporating sodium sulfate into the concrete before crushing, as referred to in the treatments in [29].

In a 90-s initial mixing process before casting, sodium sulfate was dissolved in two-thirds of the water and mixed with cement and natural coarse aggregate. Then, the fine aggregate was added to the mixture and stirred for 60 s. Lastly, to guarantee even mixing, the polycarboxylate superplasticizer was combined with the remaining one-third water for 120 s. The concrete measuring 100 mm × 100 mm × 400 mm was created and cured for 28 days.

### 3.3. No-Sulfate Recycled Aggregate and Sulfate-Containing Recycled Aggregate

Traditional RCA and SRA were obtained by crushing the parent natural concrete and sulfate-containing concrete using a crushing machine (Nanbei Instruments Ltd., Zhengzhou, China) after the concrete had finished hardening. C20, C30, and C40 concrete were used to make RCA20, RCA30, and RCA40, respectively. C20S, C30S, and C40S concrete were used to make SRA20, SRA30, and SRA40, respectively. The recycled aggregate had a particle size range of 5 to 20 mm. The apparent density, water absorption, and crushing value of RCA and SRA were tested.

### 3.4. Recycled Aggregate Treated by Cement Paste Wrapping

The cement wrapping paste approach was used to treat the RCA and SRA. The water–binder ratio and fly ash dosage were determined by referencing the exploratory experiments of earlier studies [17,19]. To mitigate excessive wrapping thickness caused by an excessively low water–binder ratio, a ratio between 0.6 and 1.0 was adopted. As demonstrated in [30], a 15% fly ash content optimally promoted secondary hydration reactions and enhanced concrete durability; this specific fly ash dosage was implemented in the current investigation. Table 3 displays the codes for different modified aggregates.

**Figure 1:**
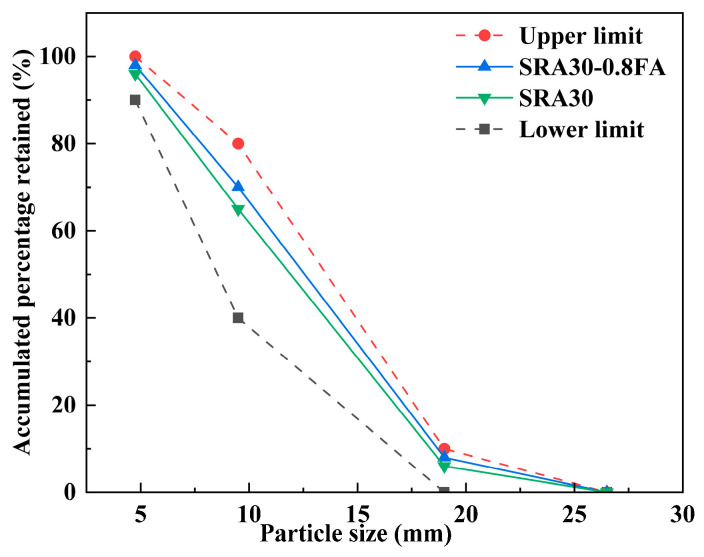
Particle size distribution curves of SRA30 and SRA30-0.8FA.

Before the wrapping process, the recycled aggregates were soaked in water until saturated, after which the surface water was wiped off. The wrapping paste was prepared by mixing cement, fly ash, and water, followed by the addition of an appropriate amount of polycarboxylate superplasticizer. The recycled aggregates were immersed in the wrapping paste and repeatedly sieved to ensure thorough and uniform coating. Figure 1 shows the particle size distribution curves of representative sulfate-containing recycled aggregate (SRA30) and modified recycled aggregate (SRA30-0.8FA). The particle size of the selected unmodified recycled aggregates was 5–20 mm. After paste wrapping, the particle size of the aggregates increased slightly, but still falls within the range of continuous gradation.

### 3.5. Test Methods

#### 3.5.1. Compressive Strength of Parent Concrete

To evaluate the effect of internal sulfate on the mechanical properties of concrete, the compressive strength of parent concrete and sulfate-containing concrete (100 mm×100 mm × 100 mm) was tested with a TYW-2000 testing machine (Sida Testing Technology Ltd., Jinan, China), referring to GB/T 50081-2019 [31]. The average value of the three samples was used to characterize the compressive strength.

#### 3.5.2. Physical Properties of Recycled Aggregate

The impact of internal sulfate on physical properties of recycled aggregate was measured. In addition, the enhancement of cement paste wrapping on the characteristics of SRA was also assessed. Owing to the adhered mortar content, recycled aggregates exhibited a lower apparent density and higher water absorption compared to natural aggregates. The physical parameters of recycled aggregates include apparent density, water absorption, and crushing value, according to GB/T 25177-2010 [27]. The apparent density of recycled aggregates was determined using the drainage method. The water absorption was defined as the ratio of the mass of absorbed water by recycled aggregate under 24-h water immersion to its oven-dried mass. The crushing value was a key mechanical performance indicator for recycled aggregates themselves. It is determined as the percentage of particles that pass through a 2.36 mm sieve after a specified compressive load for a defined duration.

#### 3.5.3. Microhardness Characteristics of Recycled Aggregate

**Figure 2:**
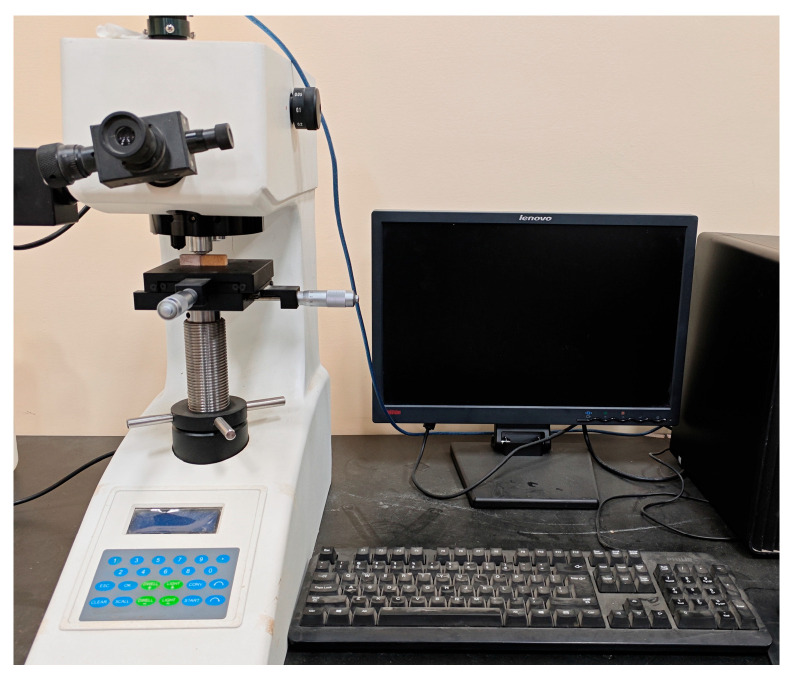
The photo of microhardness tester.

The microhardness of the recycled aggregate and interface was measured by HX-1000 T microhardness tester (Shanghai Optical Instrument Factory, Shanghai, China), according to JJG148-2006 [32]. The photo of microhardness tester is provided in Figure 2. To minimize experimental result errors, the interface was first identified using a tester camera under a low-power lens, followed by switching to a high-power lens for precise indentation positioning. The test force was set as HV0.1, and microhardness values at each point were recorded.

#### 3.5.4. Microstructure of Recycled Aggregate

The microstructure of wrapped RCA and SRA was examined using a Hitachi Regulus-8100 SEM (Hitachi, Tokyo, Japan) (×2000). Samples containing aggregate, mortar, and interface were carefully selected and prepared for analysis. In addition to SEM analysis, the pore structure of the modified aggregates was quantitatively characterized using MIP (AutoPore V 9600 porosimeter, Micromeritics instrument Ltd., Norcross, GA, USA). Prior to SEM and MIP analysis, the specimens were cleaned with ethanol to remove surface impurities and then dried in a vacuum oven at 60 °C to ensure complete moisture removal.

## 4. Results and Discussion

### 4.1. Effect of Sulfate on the Properties of Concrete and of Recycled Aggregate

#### 4.1.1. Compressive Strength of Concrete

**Figure 3:**
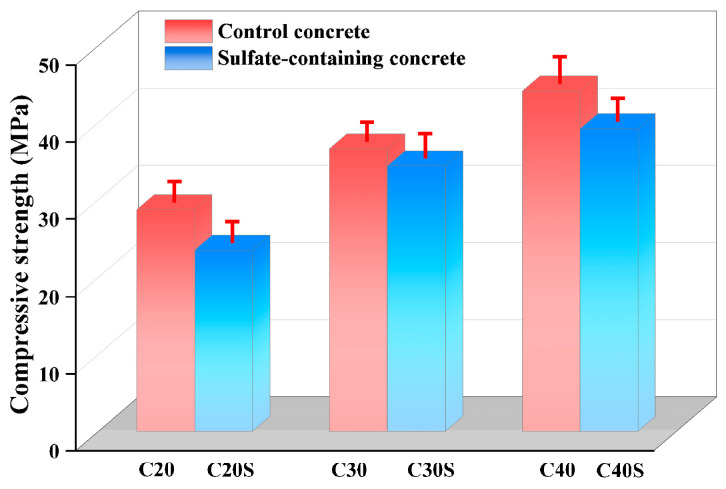
Compressive strength of control concrete and sulfate-containing concrete.

Figure 3 shows the results of compressive strength test of sulfate-containing concrete. The compressive strength of sulfate-containing concrete was lower than that of control concrete without sulfate. The 28-day cubic compressive strengths of C20, C30, and C40 concrete were 28.7 MPa, 36.6 MPa, and 44.1 MPa, respectively. These values decreased by 18.1%, 6.0%, and 11.1%, respectively, after the addition of 2% Na_2_SO_4_. It is acknowledged that the internal sulfate may react to produce gypsum or ettringite with calcium hydroxide and hydrated calcium aluminate. The increase in volume of these components generated concentrated internal stresses, which promoted crack initiation and sulfate propagation, eventually resulting in concrete expansion and structural disintegration [29].

#### 4.1.2. Apparent Density of SRA

**Figure 4:**
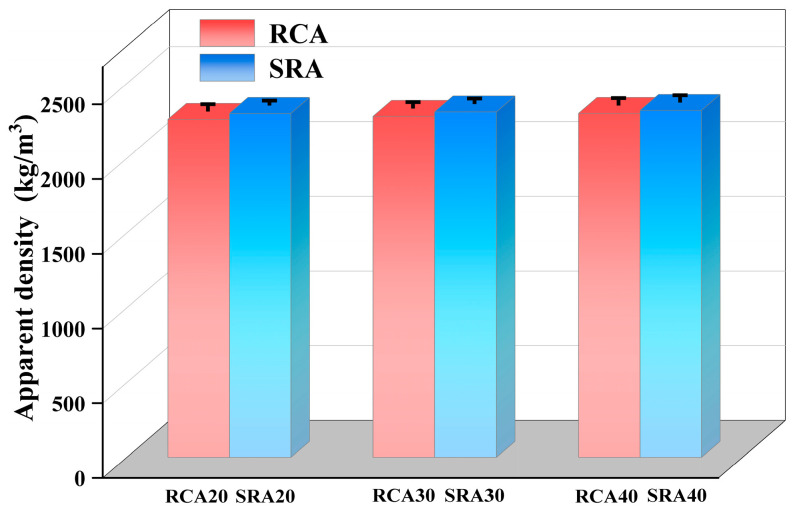
Apparent density of RCA and SRA.

The results of apparent density measurements of RCA and SRA from natural parent concrete and sulfate-containing concrete are displayed in Figure 4. The apparent densities of RCA20, RCA30, and RCA40 were 2260, 2280, and 2300 kg/m^3^, respectively. In contrast to the compressive strength results, the sulfate in the parent concrete decreased the compressive strength of concrete while increasing the apparent density of RCA. Relative to the apparent density values observed in RCA20, RCA30, and RCA40, the corresponding SRA series (SRA20, SRA30, and SRA40) exhibited density increases of 2.0%, 1.3%, and 0.8%, respectively. The compressive strength of concrete was minimally affected when sulfate content in the aggregate was below 3% [33]. When the sulfate content remained within a specific range, its impact on recycled aggregate quality became relatively insignificant. It was attributed to the crystallization and deposition of ettringite and gypsum within the micro-cracks and pores of the adhesive mortar. These crystalline products filled the initial voids, consequently leading to an observable increase in the apparent density of RCA.

#### 4.1.3. Water Absorption of SRA

**Figure 5:**
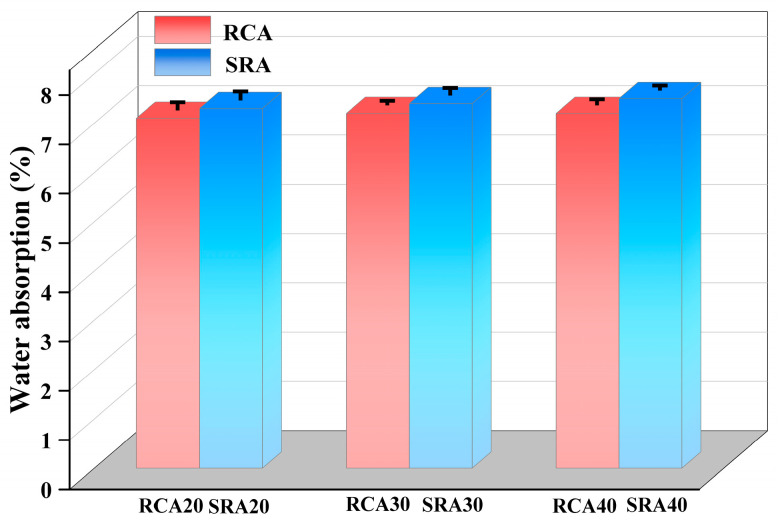
Water absorption of RCA and SRA.

The results of water absorption measurements of RCA and SRA from natural parent concrete and sulfate-containing concrete are presented in Figure 5. Comparative analysis revealed that sulfate-containing aggregates exhibited significantly higher water absorption compared to non-sulfate RCA, with absorption increments of 2.8%, 2.7%, and 4.2% for SRA20, SRA30, and SRA40, respectively. There were two possible synergistic interpretations for this increase in water absorption. Firstly, sulfate attack induced the crystallization of ettringite and gypsum within the internal pores of the aggregate and interfacial transition zones. These erosion products generated expansive stresses that compromise the structural integrity of recycled aggregate, creating additional water penetration pathways. Secondly, the newly formed ettringite and gypsum crystals possessed intrinsic hygroscopic properties, thereby amplifying the overall moisture uptake capacity of the cementitious system. Dobrovolski et al. [34] investigated the internal sulfate erosion phenomenon caused by pyrite oxidation and confirmed the nucleation expansion mechanism of gypsum and ettringite phases. Zhang et al. [35] observed that the water absorption of recycled aggregates increased significantly under severe sulfate erosion, whereas it exhibited a decrease under slight exposure.

#### 4.1.4. Crushing Value of SRA

**Figure 6:**
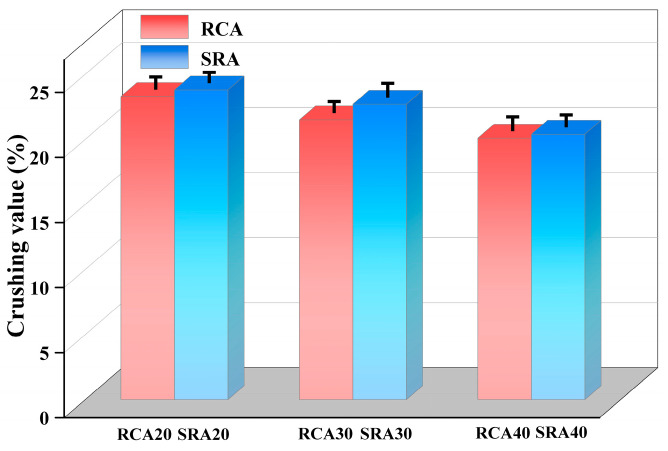
Crushing value of RCA and SRA.

The results of crushing value measurements of RCA and SRA from natural parent concrete and sulfate-containing concrete are presented in Figure 6. The crushing values of control non-sulfate RCA20, RCA30, and RCA40 were 23.3%, 21.5% and 20.1%, respectively. The crushing values of sulfate-containing SRA20, SRA30, and SRA40 were 23.8%, 22.7%, and 20.4%, respectively. It was found that the crushing value deteriorated when sulfate was internal in the parent concrete, although the apparent density of SRA increased, as shown in Figure 4. The gypsum and ettringite erosion products filled the pores of the adhesive mortar, potentially increasing its density slightly. However, these products lacked intrinsic strength, and their water absorption and expansion generated stress on capillary pore walls. This process induced micro-cracks and weakened the interface transition zone of sulfate-containing aggregates, ultimately increasing their susceptibility to crushing and the properties of crushing value. This phenomenon was also proved by Colman et al. [36], who recommended that sulfate content should be strictly limited, due to the risk of internal sulfate attack. Zhao et al. [28] researched the mechanism of internal aggressive and external attack on concrete with tailing aggregates. Regarding the internal sulfate erosion, the calcium hydroxide was consumed, and a lot of degradation products were formed. These processes also limited the C-S-H gels, therefore reducing the early strength of concrete.

### 4.2. Properties of Paste Wrapped Recycled Aggregate

#### 4.2.1. Apparent Density

**Figure 7:**
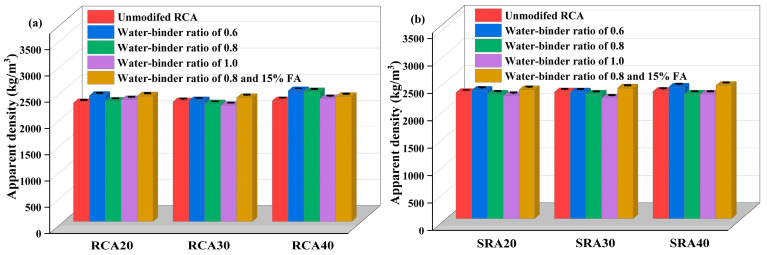
Apparent density of paste wrapped RCA (**a**) and SRA (**b**).

The apparent density of paste wrapped RCA and SRA is presented in Figure 7. Results indicated an inverse relationship between the apparent density of paste wrapped RCA and water–binder ratio. For instance, the modified RAC30-0.6, RAC30-0.8, and RAC30-1.0 specimens showed densities of 2300 kg/m^3^, 2240 kg/m^3^, and 2210 kg/m^3^, respectively, which increased by 0.88%, reduced by 1.75%, and 3.07% compared to the baseline apparent density of 2280 kg/m^3^ for RCA30 aggregate. These findings suggested that the modification efficacy of cement paste wrapping was significantly dependent on the paste proportions, with a water–binder ratio of 0.6 appearing optimal for density enhancement. The optimal water–binder ratio of 0.6 derived in the present study was distinct from the results reported by Zhao et al. [17]. Their research indicated that, within the water–binder ratio range of 0.5–1.0, the density of modified recycled aggregates exhibited an initial increase followed by a decrease, with 0.8 being the optimal ratio. Specifically, an overly thick wrapping layer leads to increased water absorption of the wrapped recycled coarse aggregate, while an overly thin layer is insufficient for the effective modification of the adhesive mortar. In the current study, an appropriate water-reducing agent was incorporated into the wrapping paste to guarantee its fluidity, thereby ensuring that the cement paste with a water–binder ratio of 0.6 does not excessively wrap the aggregate surfaces.

Notably, while the apparent density of modified RCA generally exhibited a decreasing trend with an increasing water–binder ratio, the maximum density was achieved at specimens with 0.8 w/b containing 15% fly ash. For instance, compared to the RCA30-0.8 specimen (2240 kg/m^3^), the RCA30-0.8FA specimen had a higher apparent density (2360 kg/m^3^). The secondary hydration reaction between fly ash and calcium hydroxide in adhesive mortar exerted an excellent repairing effect. Wang et al. [18] used a paste wrapping treatment with 0.5 w/b and 20% fly ash to modify recycled aggregate, which considerably increased the frost resistance of concrete. It was expected that the modifying effect of aggregates in this study would be better when a w/b of 0.6 and proper fly ash were used.

The cement paste wrapping modification laws for SRA were comparable to those for traditional recycled aggregate. Compared with SRA20, the apparent density of SRA20-0.6 increased by 1.74%, while that of SRA20-0.8 and SRA20-1.0 decreased by 1.30% and 2.61%, respectively. Tian et al. [37] investigated the internal sulfate diffusion in cement mortar with varying water–binder ratios and determined that when the water–binder ratio was less than 0.6, the internal sulfate diffusion was restricted. There were more pores in the attached mortar with a higher water–binder ratio, which increased the sulfate transport channel. In addition, Leklou et al. [38] confirmed that an increased w/b ratio exacerbated deterioration due to internal sulfate attack. Therefore, an excessively high w/b ratio restricted the potential for modifying sulfate-containing aggregates. SRA20-0.8FA possessed the highest apparent density as shown in Figure 7b. Sulfate acted as an activator for fly ash, encouraging its secondary hydration reaction and the early strength of fly-ash cementitious materials [39]. In terms of sulfate-containing SRA, fly ash can enhance the modifying impacts of cement paste wrapping.

#### 4.2.2. Water Absorption

**Figure 8:**
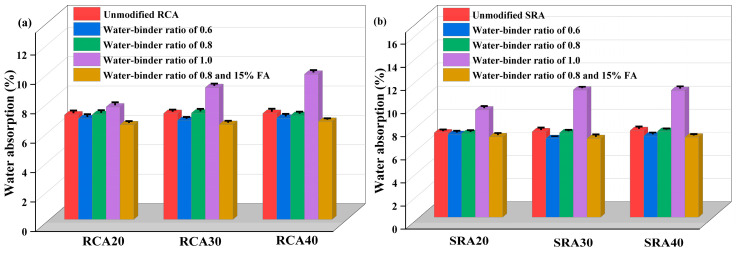
Water absorption of paste wrapped RCA (**a**) and SRA (**b**).

The water absorption of wrapped RCA and SRA is provided in Figure 8. With the increase in the water–binder ratio, the water absorption of wrapped aggregate increased. Compared to the baseline water absorption of 7.1% for unmodified RCA20, wrapped aggregate with w/b of 0.8 and 1.0 exhibited increased absorption of 7.12% and 7.64%, respectively, indicating negative effective improvement. Compared to the baseline water absorption of 7.3% for unmodified SRA20, wrapped aggregate with a w/b of 1.0 showed much greater water absorption (9.28%). When the w/b was 0.6, the enhancement effects of cement paste wrapping were observed for SRA20-0.6, with water absorption of 7.16%. A higher w/b induced looser pore structures in the paste, thereby enhancing its water absorption capacity. This behavior was proved by Wang et al. [18] who found that recycled aggregate treated with 0.5 w/b had a better effect on the frost resistance of concrete. However, this finding contrasted with Zhang et al. [40], where the water absorption of cement paste wrapped aggregate with a w/b of 0.8 was higher than that with a ratio of 1.0. This phenomenon may be caused by the high consistency and thick coating of the paste with a higher water–binder ratio. Even so, their experimental findings also revealed that the durability performance of recycled concrete with a 0.8 water–binder ratio wrapping was greatly improved, showing a considerable restorative effect of the cement paste wrapping approach on the recycled aggregate interface. In our experiment, water-reducing chemicals were used to provide the paste with great fluidity without compromising its structure.

The optimum technique to wrap both traditional recycled aggregate and sulfate-containing recycled aggregate was to use paste with 0.8 w/b and 15% fly ash. For instance, this coating reduced aggregate water absorption by 2.9% and 7.7%, respectively, as compared to RCA40 and SRA40. Experimental results demonstrated that cement-based cementitious paste exhibited superior performance enhancement on SRA compared to traditional RCA. Zhang et al. [41] validated this finding through systematic testing of paste-treated recycled aggregates, confirming its functionality in blocking sulfate ion penetration and enhancing the external sulfate resistance of recycled concrete. Moreover, the supplementary wrapping paste contributed to resisting the tensile stress caused by sulfate. As shown in Figure 8, given the superior modified effects of fly ash-based paste wrapping on SRA, Singh et al. [42] reported comparable findings. By encapsulating recycled aggregates with pozzolanic paste, they observed concurrent improvements in the mechanical properties of the adhesive mortar and a reduction in its water absorption capacity. In addition, fly ash helped create the dense interface layer and trigger its pozzolanic activity by capturing the leaky sulfate from aggregate [43].

#### 4.2.3. Crushing Value

**Figure 9:**
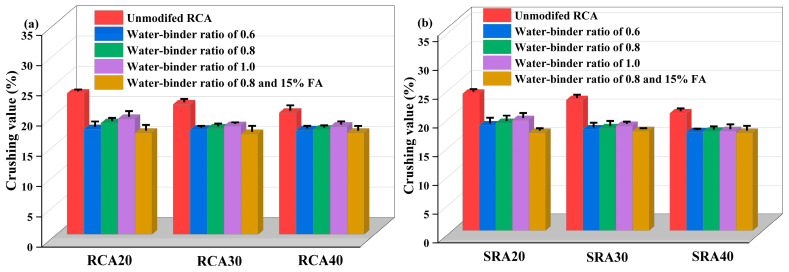
Crushing value of paste wrapped RCA (**a**) and SRA (**b**).

Figure 9 shows the crushing value of paste wrapped RCA and SRA. Different from the results of apparent density and water absorption (where only the wrapped paste with 0.6 w/b was effective), all the wrapped paste mixtures adopted in the experiment reduced the aggregate crushing value. This result contradicted the findings reported by Zhang et al. [41], who observed an increase in the crushing value when using paste wrapping technology. Their study involved curing the wrapping paste for only 3 days, leaving it more susceptible to damage during the crushing value test. In contrast, our research utilized a 28-day curing period, which allowed the paste to develop sufficient strength, ultimately contributing to the reduced crushing value of both RCA and SRA.

Additionally, the crushing value of paste wrapped RCA and SRA increased with w/b (0.6–1.0), which was consistent with the apparent density results. Adding 15% fly ash to the paste with a w/b of 0.8 reduced the RCA and SRA’s crushing value further. Taking RCA30 and SRA30 aggregates as examples, the wrapping paste with a 0.8 w/b and 15% fly ash demonstrated the best performance, lowering their crushing values by 24.1% and 23.7%, respectively. This matches the findings of Chen et al. [44], who reported that treating recycled concrete and brick aggregates with fly ash-based geopolymer paste resulted in a crushing value reduction of over 20%. Further decreasing the w/b and adding fly ash or other mineral admixtures may result in a more significant modifying impact. Yang et al. [45] found that using a 0.3 w/b and 5% silica fume improved the performance of recycled aggregate and concrete, but an adequate water-reducing agent had to be added to assure fluidity and coating thickness. The best proportions for cement paste wrapping modification of aggregates require additional examination.

### 4.3. ANOVA Analysis

To examine whether significant differences exist in the mean values among the samples, a one-way analysis of variance (ANOVA) was employed to assess the statistical significance of the results between unmodified (Figure 4, Figure 5 and Figure 6) and modified aggregates (Figure 7, Figure 8 and Figure 9). The results are presented in Table 4. In terms of the unmodified recycled aggregate, the apparent density (F = 3.37, *p* = 0.039) and crushing value (F = 22.26, *p* < 0.001) of the various aggregates varied significantly, with the crushing values differing more than the apparent density. The crushing value is an indicator of aggregate strength and is more important for evaluating aggregate performance. However, it was found that the difference in water absorption among unmodified aggregates was not significant. This could be because the strength differences between the parent concretes (C20–C40) were minor, and the sulfate concentration (2%) in the parent concrete was at the minimum limit of GB/T 25177-2010 [27]. Nevertheless, there were significant differences among apparent density (F = 28.32, *p* < 0.001), water absorption (F = 76.04, *p* < 0.001), and crushing value (F = 78.83, *p* < 0.001) of the modified aggregates, as shown in Table 4. It indicated the reliability of the results of the cement paste wrapping method in improving the quality of aggregates.

### 4.4. Microhardness

**Figure 10:**
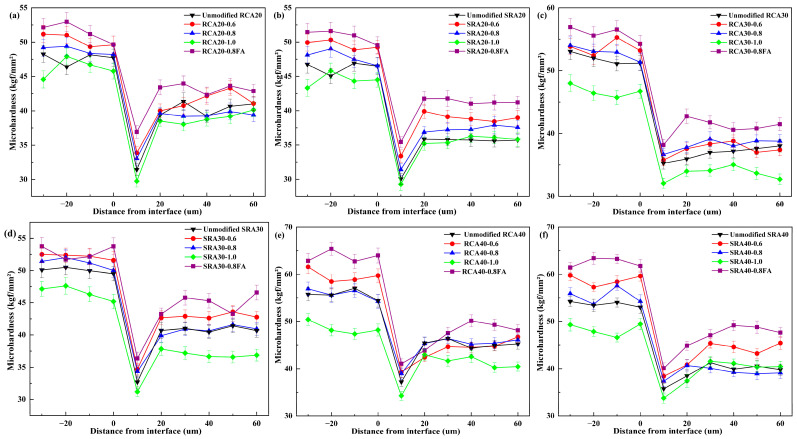
The microhardness of different modified aggregates: RCA20 (**a**), SRA20 (**b**), RCA30 (**c**), SRA30 (**d**), RCA40 (**e**), SRA40 (**f**).

Figure 10 illustrates the results of microhardness tests of each sample, where each data point denotes the average measurements of five points. The microhardness chart can be divided into three parts, namely, aggregate, interface, and adhesive mortar. The microhardness ranges of aggregates, interfaces, and mortar were respectively between 42 and 63 kgf/mm^2^, 29 and 42 kgf/mm^2^, and 33 and 48 kgf/mm^2^ for modified RCA and SRA. It was obvious that the interface was the weakest area of the recycled aggregates. In contrast to RCA20 and SRA20, the paste of 0.6 w/b exhibited a 7.76% and 11.0% enhancement in interface microhardness, and the paste of 0.8 w/b led to a 5.15% and 4.46% increase. Significantly, the paste of 0.8 w/b containing 15% fly ash demonstrated a more pronounced improvement (17.4% and 17.8%), while the paste of 1.0 w/b resulted in a 2.48% and 5.34% reduction in interfacial microhardness. It is recommended to adopt a smaller water–binder ratio and add 15% fly ash to the wrapping paste.

**Figure 11:**
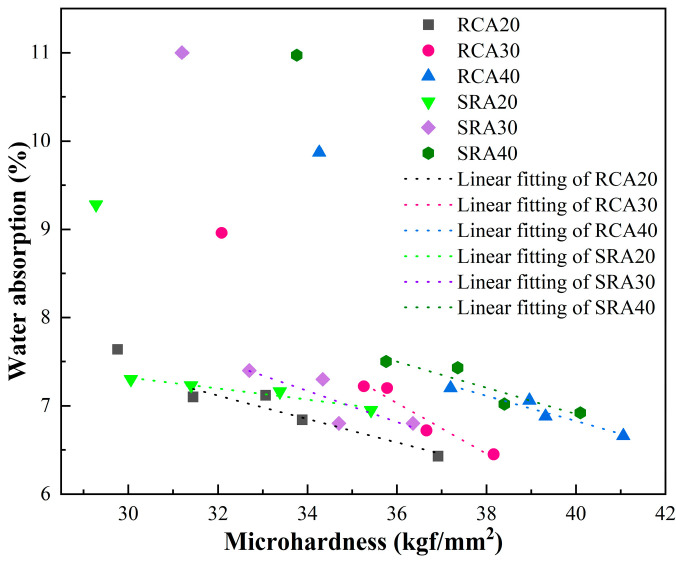
The relationship between interfacial microhardness and water absorption of modified recycled aggregates.

In general, the interfacial characteristics of recycled aggregates are associated with their quality parameters. Figure 11 illustrates the relationship between interfacial microhardness and water absorption of modified recycled aggregates. With the exception of one anomaly, it revealed a linear decrease in water absorption with increasing microhardness. The measured increase in aggregate micro hardness clearly indicated that the paste wrapping achieved better interface repair results, and this improvement was further evidenced by the decreased water absorption of the treated aggregate. This anomaly corresponded to recycled aggregates treated with paste at a w/b of 1.0. This type of cement paste exhibited high water absorption itself, resulting in abnormally high water absorption for the treated aggregates, and thus failing to improve aggregate quality.

### 4.5. SEM

**Figure 12:**
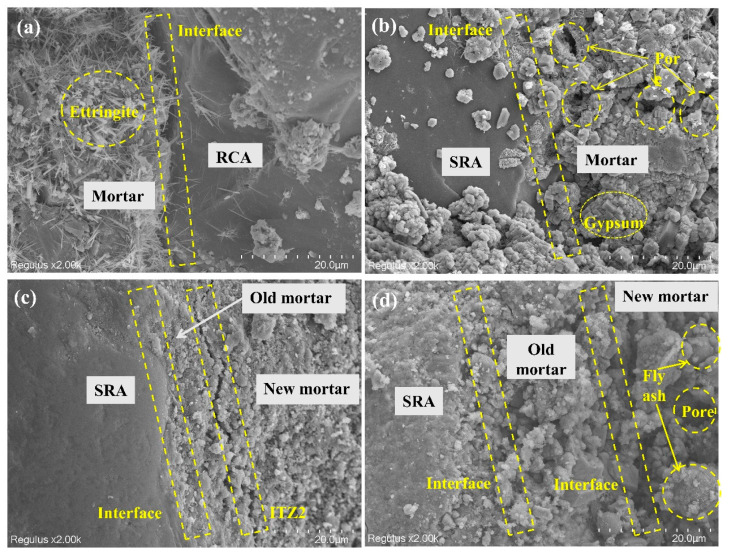
The microscopic morphology of recycled aggregate: RCA30 (**a**), SRA30 (**b**), SRA30-0.6 (**c**), SRA30-0.8FA (**d**).

The microscopic morphology of recycled aggregate is shown in Figure 12. Figure 12a depicts the ordinary recycled aggregate RCA30. Numerous needle-like crystals were found in the interfacial adhesive mortar, which was the ettringite hydration product. The migration and enrichment of calcium ions and aluminum ions at interfaces in the cement-based materials promoted the preferential formation and accumulation of ettringite in these zones [46,47]. Figure 12b demonstrates the sulfate-containing recycled aggregate SRA30. Its bonding mortar contained a lot of gypsum block crystals, and the gypsum itself was surrounded by numerous pores and cracks. The addition of sodium sulfate to the adhesive mortar resulted in the formation of gypsum [48]. According to studies [49], excessive amounts of sulfate caused gypsum rather than ettringite to be the primary erosion products in cement-based materials. In sulfate-induced erosion, sulfate predominantly accumulated within the interface in the form of gypsum [50]. Figure 12c shows the original old mortar and new wrapped paste surrounding the aggregates. The original interface width between the recycled aggregates and the old mortar was reduced and repaired by applying a cement coating with a w/b of 0.6. This confirmed that the cement paste wrapping method had a significant promotion effect on aggregate performance. However, the newly introduced cement paste created an extra interface, limiting its promotion effect. This observation was similar to Panghal et al.’s [51] findings, demonstrating that cement paste treatment of RCA effectively filled pores, thereby enhancing its strength. Nevertheless, while forming a more complex interface, the paste treatment showed limited effectiveness in reducing water absorption. Figure 12d shows the sulfate-containing aggregates modified with 20% fly ash and a 0.8 w/b (SRA30-0.8FA) are depicted morphologically. Spherical fly ash was observed in Figure 12d, and their secondary hydration contributed to interface enhancement [52,53].

### 4.6. MIP

**Figure 13:**
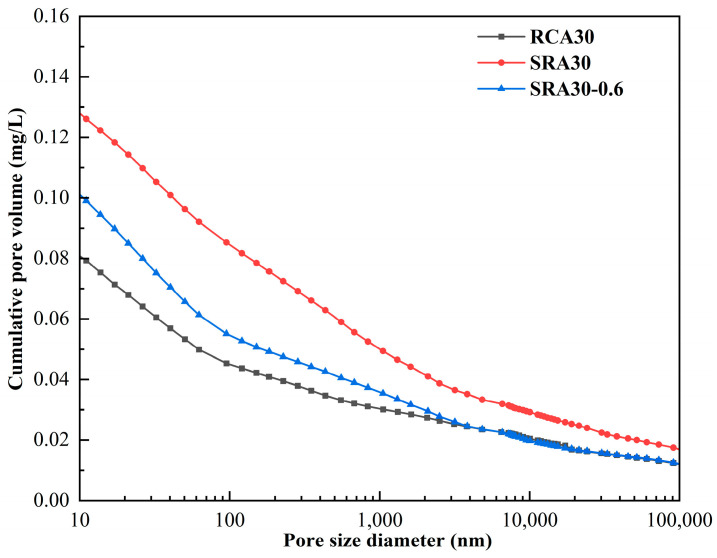
The cumulative pore volume of recycled aggregates.

The pore structure of aggregates is a key factor in describing their physical and mechanical properties. By using MIP analysis, the primary pore structure parameters of the ordinary recycled aggregate (RCA30), sulfate-containing recycled aggregate (SRA30), and modified sulfate-containing recycled aggregate (SRA30-0.6) were identified, as indicated in Table 5. Figure 13 shows the cumulative pore volume of recycled aggregates. The unmodified RCA30 displayed porosity and total pore area of 16.7% and 12.78 m^2^/g in Table 5, while the unmodified SRA30 with internal sodium sulfate showed a significant increase with values of 23.3% and 20.51 m^2^/g. Furthermore, the medium pore diameter of SRA30 (219.32 nm) was significantly larger than that of RCA30 (94.82 nm). The internal addition of sulfate results in expansion products such as ettringite, which weaken the pore structure, increase porosity, and induce cracks.

The porosity of the SRA30 decreased after being wrapped with cement paste at a w/b of 0.6. Additionally, the median pore diameter of SRA30-0.6 was significantly lower than that of SRA30. After wrapping treatment of the sulfate-containing recycled aggregates, a significant amount of C-S-H is produced by cement hydration. These materials fill the internal micro-cracks and holes and reinforce the weak interface of the aggregate. In Table 5, the modified SRA30-0.6 exhibited higher porosity but significantly smaller median pore diameter compared to the RCA30. Although the performance of sulfate-containing aggregates treated with cement paste was not entirely equivalent to that of conventional sulfate-free recycled aggregates, this treatment significantly improved their pore structure characteristics. Permeability serves as an indicator of a material’s ability to permit fluid passage. When recycled aggregates are incorporated into concrete, permeability can be used to assess the recycled concrete’s durability. The minimal permeability observed in the SRA30-0.6 specimen provides additional evidence for the effectiveness of the cement paste coating technique.

In Figure 13, SRA30 exhibited the highest cumulative pore volume, demonstrating greater porosity than both RCA30 and SRA30-0.6. Aggregate porosity increased as a result of internal sodium sulfate, necessitating modification. The SRA30-0.6 aggregate wrapped paste with 0.6 w/b had a higher cumulative pore volume than the RCA30. However, there was almost no variation in the cumulative pore volume curves in the >5000 nm region. This observation clearly proved the efficacy of cement paste wrapping in reducing macro-pores in sulfate-containing aggregates. Nevertheless, the porosity of the cement paste itself also introduced micropores, resulting in a limited reduction effect of overall porosity.

## 5. Conclusions

1. A 2% sulfate addition to the parent concrete resulted in strength decrease. Furthermore, the corresponding sulfate-containing recycled aggregate showed a decrease in crushing value and water absorption rate, whereas its apparent density increased.
2. The performance of traditional recycled aggregates and sulfate-containing recycled aggregates can be optimized by adopting an appropriate cement paste proportion. Their performance showed a decreasing trend with the increase in the water–binder ratio from 0.6 to 1.0. Notably, the water–binder ratio of 1.0 induced a notable deterioration in aggregate performance compared to unmodified aggregate.
3. The optimal wrapping paste proportion identified in this study was a 0.8 water–binder ratio and 15% fly ash, which modified sulfate-containing aggregates more effectively than traditional recycled aggregates. Considering the superior wrapping performance of a 0.6 water–binder ratio over 0.8, decreasing the water–binder ratio further and adding fly ash may lead to a more substantial modifying effect.
4. The interface of sulfate-containing recycled aggregates was primarily composed of gypsum crystals. Cement paste wrapping significantly improved the old interface structure, despite a new interface being observed in the modified aggregates.

Overall, the cement paste wrapping treatment effectively improved the quality of sulfate-containing recycled aggregates when the water–binder ratio was between 0.6 and 0.8. Additionally, incorporating 15% fly ash in the paste is recommended for optimal results.

Notably, it is important to point out that the current research is limited to a small-scale evaluation of aggregate properties. Long-term durability must be considered for field large-scale applicability, such as carbonation and sulfate attack. Although the use of cement paste wrapping can enhance the physical and mechanical properties of sulfate-containing aggregates themselves, the sulfate leaching issue warrants consideration in the long-term service life. This is the most crucial issue to be noted in the field-scale application of sulfate-containing recycled aggregates.

## Figures and Tables

**Table 1:** Chemical compositions of cement and fly ash (%).

Types	SiO_2_	Al_2_O_3_	Fe_2_O_3_	MgO	CaO	K_2_O	SO_3_
Cement	22.06	6.47	4.53	1.43	60.43	0.42	1.86
Fly ash	43.96	31.14	9.16	1.01	9.01	2.03	0.73

**Table 2:** Raw material consumption of concrete (kg/m^3^).

ID	Cement	Coarse Aggregate	Fine Aggregate	Water	SP	Na_2_SO_4_
C20	408	821	778	212	2.04	-
C20S	408	821	778	212	2.04	8.14
C30	460	858	735	196	2.30	-
C30S	460	858	735	195	2.30	9.17
C40	503	890	700	182	2.51	-
C40S	503	890	700	181	2.51	10.05

**Table 3:** The codes for different modified recycled aggregates with wrapped paste proportions.

Codes	w/b = 0.6	w/b = 0.8	w/b = 1.0	w/b = 0.8, 15% Fly Ash
RCA20	RCA20-0.6	RCA20-0.8	RCA20-1.0	RCA20-0.8FA
RCA30	RCA30-0.6	RCA30-0.8	RCA30-1.0	RCA30-0.8FA
RCA40	RCA40-0.6	RCA40-0.8	RCA40-1.0	RCA40-0.8FA
SRA20	SRA20-0.6	SRA20-0.8	SRA20-1.0	SRA20-0.8FA
SRA30	SRA30-0.6	SRA30-0.8	SRA30-1.0	SRA30-0.8FA
SRA40	SRA40-0.6	SRA40-0.8	SRA40-1.0	SRA40-0.8FA

**Table 4:** ANOVA results of unmodified and modified recycled aggregate.

Properties	Unmodified Recycled Aggregate		Modified Recycled Aggregate	
	F-Value	*p*-Value	F-Value	*p*-Value
Apparent density	3.37	0.039	28.32	<0.001
Water absorption	2.03	0.145	76.04	<0.001
Crushing value	22.26	<0.001	78.83	<0.001

**Table 5:** Relevant parameters of the pore structure of recycled aggregates.

Pore Structure Parameter	RCA30	SRA30	SRA30-0.6
Porosity (%)	16.7%	23.3%	20.8%
Total pore area (m^2^/g)	12.78	20.51	18.18
Medium pore diameter (nm)	94.82	219.32	75.73
Permeability (mdarcy)	355.4	641.4	262.1

## Data Availability

The original contributions presented in this study are included in the article. Further inquiries can be directed to the corresponding author.
